# Personalized Media: A Genetically Informative Investigation of Individual Differences in Online Media Use

**DOI:** 10.1371/journal.pone.0168895

**Published:** 2017-01-23

**Authors:** Ziada Ayorech, Sophie von Stumm, Claire M. A. Haworth, Oliver S. P. Davis, Robert Plomin

**Affiliations:** 1 King's College London, MRC Social, Genetic and Developmental Psychiatry Centre, Institute of Psychiatry, Psychology & Neuroscience, London, United Kingdom; 2 Department of Psychology, Goldsmiths University of London, London, United Kingdom; 3 MRC Integrative Epidemiology Unit, School of Experimental Psychology & School of Social and Community Medicine, University of Bristol, Bristol, United Kingdom; 4 MRC Integrative Epidemiology Unit, School of Social and Community Medicine, University of Bristol, Bristol, United Kingdom; University of Warwick, UNITED KINGDOM

## Abstract

Online media use has become an increasingly important behavioral domain over the past decade. However, studies into the etiology of individual differences in media use have focused primarily on pathological use. Here, for the first time, we test the genetic influences on online media use in a UK representative sample of 16 year old twins, who were assessed on time spent on educational (N = 2,585 twin pairs) and entertainment websites (N = 2,614 twin pairs), time spent gaming online (N = 2,635 twin pairs), and Facebook use (N = 4,333 twin pairs). Heritability was substantial for all forms of online media use, ranging from 34% for educational sites to 37% for entertainment sites and 39% for gaming. Furthermore, genetics accounted for 24% of the variance in Facebook use. Our results support an active model of the environment, where young people choose their online engagements in line with their genetic propensities.

## Introduction

People aged 16 to 24 years are among the most extensive users of digital, social, and mobile technology in the UK (National Statistics http://www.ons.gov.uk/ons/dcp171778_404497.pdf). Online media use, including web surfing, gaming and social networking, has become a common part of daily life, with almost 90% of all UK households having access to the Internet (National Statistics http://www.ons.gov.uk/ons/dcp171778_373584.pdf). Given the growth in availability of media use, online and off, researchers have increasingly been interested in the consequences of the use of such media. In some cases, such as the influence of violent media [1] and media depicting sexualized content [2]—influences appear to be minimal. In other areas such as sleep disturbances [3], sedentary behavior [4] or antisocial behavior [5, 6], correlations appear to exist, although causality can be difficult to determine.

Recently, the psychological literature has also begun to recognize the potential benefits of online media, which have permeated many aspects of our social, educational and occupational lives [7]. This development calls for an investigation of the origin of individual differences in online media use, especially as all previous studies in this area focused on clinical samples and on problematic or pathological internet use [5, 6, 8–10] or were based on small sample sizes [11].

Ubiquitous genetic influence is now widely accepted for nearly all psychological traits [12]; it is therefore reasonable to predict that online media use would also show genetic influence. Finding a role of genetics on an ostensibly ‘environmental’ measure would challenge the passive view of media effects models [13, 14] and instead support an active view of the media environment where individuals tailor their online media use based on their own unique genetic propensities—a concept known as *gene-environment correlation*.

Genetic mediation of the association between media use and later adult outcomes has been demonstrated in two independent samples [5, 6] both of which focussed on television viewing and later adult criminal or antisocial behavior. Together, these works suggest that unmeasured genetic factors can account for the observed associations between media use and behavioral outcomes. We therefore expected to find substantial genetic influence on individual differences in online media use in our sample.

Using a genetically informative design, we explored here for the first time individual differences in non-pathological online media use in a well powered and UK representative sample of 16-year old twins. Specifically, we investigated the aetiology of time spent on educational and entertainment media online as well as the heritability of Facebook use, the most popular social network among young people today [15].

## Materials and Methods

### Participants

The sample was drawn from the Twins Early Developmental Study (TEDS), a UK-representative sample of twins born in England and Wales between 1994 and 1996. Of the 16,000 twin pairs originally recruited, over 10,000 remain actively involved in TEDS today [16]. All individuals with self-report measures of media use, available at age 16, were included in the present study. Participants with severe medical or psychiatric problems or whose mothers had severe medical complications during pregnancy were excluded from the analysis. We also excluded participants with unknown zygosity. Zygosity was assessed by a parent-reported questionnaire of physical similarity, which is over 95% accurate when compared to DNA testing [17]. For cases where zygosity was unclear from this questionnaire, DNA testing was conducted.

Sample sizes for each of the online media use measures differed as data were collected both online and using paper questionnaires. Sample sizes were larger for the Facebook measures as they were included as part of a wider behavioral study. Data on use of educational sites were available for 5,221 individuals, of whom 1,978 were monozygotic (MZ) twin pairs, 1,674 were dizygotic same-sex (DZss) twin pairs and 1,569 were dizygotic opposite-sex (DZos) twin pairs. Data on entertainment sites were available for 5,181 individuals after exclusions, 1,961 MZ twin pairs, 1,663 DZ twin pairs and 1,557 DZos twin pairs. Time spent on online gaming was assessed in 5,273 individuals, with twin data available for 1,996 MZ pairs, 1,686 DZss pairs, and 1,591 DZos pairs. Facebook data were available for the largest number of individuals (N = 8,648); including 3,090 MZ and 2,816 DZss twin pairs and 2,742 DZos twin pairs. King’s College London Ethics Committee (PNM/09/10-104) granted project approval and written parental consent was obtained prior to data collection.

### Measures

Media use data were collected by an online questionnaire. Participants indicated on a 4-point Likert scale ranging from never (0) to more than four hours per week (3) how often they used their home computer for playing either educational or entertainment games, word processing, email, chat rooms or reading online. Facebook use was assessed by a paper questionnaire, mailed to each participant. Participants indicated whether or not they had a Facebook account, and if so how long they have had the account, ranging from less than one month (1) to five or more years (5); how often they checked their Facebook updates, ranging from three or more times per day (1) to less than once a month (7); how much time they spend on Facebook per week, ranging from less than 30 minutes (1) to 20 hours or more (6), and how many Facebook friends they had. The online media and Facebook use questionnaires appear in the Supplementary Online Material as S1 and S2 Figs.

### Analysis

#### Phenotypic

Because the eight online media use items and the four Facebook use items were from different measures with varying sample sizes, we factor analyzed the two scales separately using varimax rotation. Factors with Eigenvalues above 1 and items with loadings greater than .50 were retained.

Analysis of variance (ANOVA) tested the main effect of sex, zygosity, and their interaction on the obtained factors. Subsequently, the latter were corrected for age and sex differences using the regression method, as is standard in twin analysis [18]. Because some of the media use variables were negatively skewed, data were mapped on to a standard normal distribution using the rank-based van der Waerden’s transformation [19]. Standardized age- and sex-corrected residuals were used for all subsequent analyses.

#### Twin design

The twin design compares siblings of known genetic relatedness to estimate the proportion of phenotypic variance that can be attributed to genetic and environmental factors (Plomin, DeFries, Knopik, & Neiderhiser, 2016). MZ twins share 100% of their genes while DZ twins, like non-twin siblings, share on average 50% of the genetic material that can differ between individuals (segregating genes). Heritability (A) is narrowly defined as the proportion of individual differences in a population that can be attributed to inherited DNA differences and is estimated by doubling the difference between MZ and DZ twin correlations. Environmental contribution to phenotypic variance is broadly defined as all non-inherited influences that are shared (C) and unique (E) to twins growing up in the same home. Shared environmental effects (C) are calculated by subtracting A from the MZ twin correlation and contribute to similarities between siblings while non-shared environmental effects (E) are those experiences unique to members of a twin pair that do not contribute to twin similarity. The E component also includes measurement error and is calculated by deducting the A and C components from unity, as the total variance explained cannot exceed 100% [20].

The ACE estimates can be calculated more precisely using structural equation modeling with maximum likelihood estimation, which also provides 95% confidence intervals. Structural equation modeling (SEM) leverages the different sources of sibling similarity and differences to make inferences on the etiology of observable traits. SEM tests hypotheses about relations among observed phenotypic correlations and latent genetic and environmental factors by modeling the observed covariance between MZ and DZ twin pairs on the phenotype. Estimates of the latent factors are then based on maximum likelihood criterion which seeks to obtain the best fit for the model—the smallest possible discrepancy between the model and the observed data [20, 21].

When data are available for both DZ same-sex and DZ opposite sex twins, this standard univariate analysis can be extended to a sex-limitation model, which tests for quantitative and qualitative sex differences in the etiology of phenotypic differences. Quantitative differences estimate the magnitude of genetic and environmental effects between genders while qualitative differences test whether or not the same genes or shared environmental experiences influence males and females—which is suggested when the phenotypic correlation between DZ opposite sex twins is smaller than for DZ same sex twins. Using a series of models that are hierarchically related, sex-limitation model fitting compares nested models where the parameters are either fixed or free to vary between sexes. The relative fit of each alternative model is then tested using standard chi-squared difference tests. A non-significant chi squared value indicates that the model is consistent with the data, whereas a significant chi squared value indicates that the model provides a poor fit to the data and can be rejected. The following models were tested in our analyses: Full heterogeneity model, heterogeneity model, and homogeneity model.

In the full model, all parameters are allowed to vary across all five zygosity groups (MZ male pairs and female pairs, same-sex DZ male pairs and female pairs, and opposite-sex DZ pairs). Here we model a set of ACE parameters for males and females separately to estimate the correlation between genetic factors across genders and common environmental factors across genders using same-sex twin pairs as well as opposite sex pairs. We then test a heterogeneity model by constraining the genetic or shared environmental correlation to expected values (0.5 or 1.0 respectively), while allowing other estimates to vary. Fit statistic comparisons between the full heterogeneity and the heterogeneity model indicate whether constraining the correlations to expected values significantly reduces the fit of the data, which would indicate qualitative sex differences exist for these data. Finally, we test for quantitative sex differences by comparing the heterogeneity model to a homogeneity model. The homogeneity model is a reduced model that assumes no heterogeneity so ACE estimates are equated for both genders and the DZos genetic correlation is constrained to 0.5. If equating estimates across genders produces a worse fit for the data compared to the heterogeneity model where they are free to vary, then quantitative sex differences are suggested. The sex limitation model generates its estimates on the basis of the raw (absolute) variance. It then creates the proportional (relative) estimates by dividing the absolute values by the total variance (hence the three ACE scores summing to 1.0). Each estimate is then rounded separately to 1 or 2 decimal places, which may cause the sum of the ACE scores to deviate slightly from 1.0. The program OpenMX was used for univariate and sex-limitation model-fitting analyses [22].

## Results

### Descriptive statistics

Three factors for online media use emerged that accounted for 55% of the variance, including ‘educational screen time’, ‘entertainment screen time’ and ‘online gaming’. A single Facebook factor accounted for 46% of the variance in social networking (for factor solutions see S2 and S4 Tables). The educational factor included time spent on websites for school, word processing, email and online reading while the entertainment screen time factor included internet use for fun and time spent on chat rooms. The online gaming factor was specific to games played on the computer whether they are for educational or entertaining purposes. Composites were created for these three screen time measures and for Facebook use. All subsequent phenotypic and genetic analyses were performed on these composites only.

S1 Table in the Supplementary Online Material lists samples sizes, means, and standard deviations for all online media use variables separately for males, females and zygosity groups. Sex, zygosity and their interaction showed minimal effects on our sample, accounting for less than 1% of the phenotypic variance, except for time spent on gaming online where they explained 7% of the variance.

Females reported higher educational screen time and Facebook use, while males spent more time gaming online. The MZ twin correlations were slightly higher in females for educational screen time (0.43 females; 0.39 males) and conversely higher in males for online gaming (0.43 males; 0.36 females); however, these estimates' confidence intervals overlapped. Overlapping confidence intervals were also observed when comparing same sex with opposite sex DZ twin pairs for all media use measures as well as when the ACE estimates were compared separately for males and females.

### Univariate genetic analyses

When sex differences were tested formally using SEM, results indicated no qualitative differences but that quantitative sex differences were present for most screen time measures. Females showed slightly higher heritability estimates for rates of entertainment screen time, while males were slightly higher for educational screen time and gaming.

The finding of quantitative sex differences would suggest that the full sex-limitation model should be used to derive ACE estimates separately for males and females. However, the differences between the heritability estimates for males and females are small (e.g., 35% vs. 36% for entertaining screen time, respectively), with overlapping confidence intervals for nearly all of our measures, except gaming where confidence intervals were slightly non-overlapping (see S6 Table in Supplementary Online Material). Despite being statistically significant these sex-differences are slight and may not be significant with smaller samples. For this reason, we chose the most parsimonious model for these data, with ACE parameter estimates and variance equated between males and females. Subsequent analyses were performed on transformed, age- and sex-regressed variables, as described in the Methods section using the whole sample, combining data from DZss and DZos twin pairs. The model-fitting results and sub-model comparisons for the sex-limitation analyses are in the supplementary material as S7–S10 Tables. A detailed description of modeling sex differences in the TEDS sample has been described elsewhere [23].

**Fig 1:**
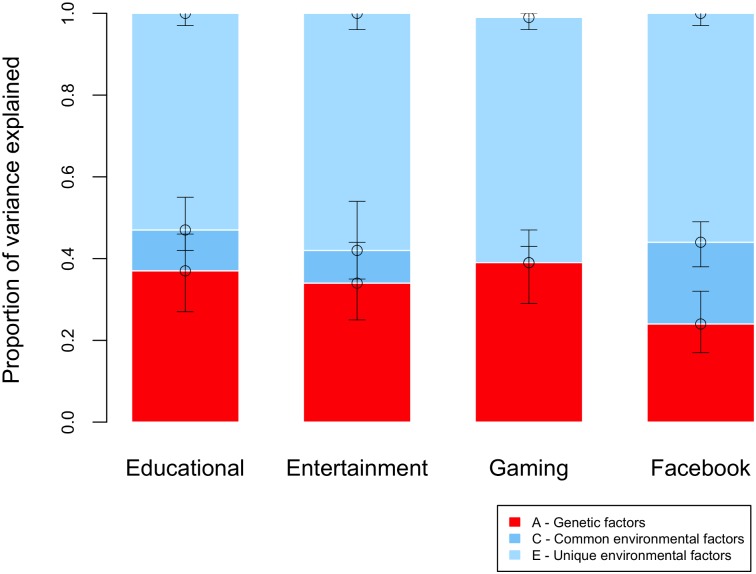
ACE results for four kinds of online media use. *Note*. Entertain = time spent on entertaining media, Educate = time spent on educational media, Gaming = time spent on online games for fun or for educational purposes, Facebook = level of engagement with the social network Facebook. Results indicate the additive genetic (A), shared (C) and non-shared (E) environmental components of variance. Estimates with 95% confidence intervals, are available in S11 Table of the Supplementary Online Material.

Genetic differences contributed substantially to individual differences in media use (Fig 1). Heritability estimates were significant for time spent on websites for entertainment (37%), educational purposes (34%), time spent playing games online (39%), and for the degree of use of the social networking site Facebook (24%). Shared environmental factors accounted for less than 10% of the variance in online media use, except for measures of Facebook use where they explained 20% of the variance. The remaining variance in media use was explained by environmental factors that do not contribute to twin similarity (including error of measurement), accounting for 53–60% of individual differences across online media use measures.

## Discussion

In the largest representative twin sample with online media use data available to date, we showed a significant genetic contribution to individual differences in online media use. Genetic influences were substantial for time spent on all types of media including entertaining (37%) and educational (34%) media, online gaming (39%) and even social networking (24%).

What is novel about the present study is that we elucidate the relative genetic and environmental contribution to a wide range of media use variables, including not only those forms of online media that have been traditionally viewed as problematic (i.e., gaming, social networking, or entertainment sites) but also online media used for educational activities.

Nearly two-thirds of the variance in online media use was explained by unique environmental factors that do not contribute to similarities between members of a twin pair growing up in the same household. Similarly large contributions of the unique environment have been reported elsewhere for compulsive Internet use (52%;[10]), hours spent online (59%;[9]) and time spent on social networking sites (57%;[9]). Our current study adds to these results that converge on one of the most consistently replicated findings in behavioral genetics, that the most salient environmental influences are those specific to each child in the family [24].

For several of our screen time measures we found evidence for quantitative sex differences suggesting that the relative contribution of genetic variation to time spent online differs as a function of gender. We note that we tested quantitative sex differences with respect to absolute variances. In these models, relative genetic variances (i.e., A effects) had overlapping confidence intervals in males and females. Therefore, we concluded that although absolute variances differed in males and females there were no sex differences in the A effects.

Evidence for sex differences in genetic aetiology is inconsistent across the literature on media use [8–11]. Where sex differences are found [8, 10], they are typically small with overlapping confidence intervals, as in the present study. That said, we note that in the current study for gaming only, the difference in the genetic effects between males and females was significant with marginally non-overlapping confidence intervals. Although TEDS is well powered to detect even small sex differences, we suggest a cautious interpretation of this finding until replication in equally well-powered independent samples has been conducted.

### Strengths and limitations

Our results provide the first well powered evidence of genetic influence on non-pathological online media use. Finding genetic effects on an ostensibly ‘environmental’ measure of media use supports our hypothesis of *gene-environment correlation* as a driving force for individual differences in media use. Gene-environment correlation refers to genetic influence on environmental exposure. The key component of this correlation is choice such that individuals are not simply passive recipients of their environment but instead actively select their experiences and these selections are correlated with their genetic propensities [25]. Our findings challenge popular media effects theories, which typically view the media as an external entity which has some effect (either good or bad) on helpless consumers [13]. Finding that DNA differences substantially influence how individuals interact with the media puts the consumer in the drivers seat, selecting and modifying their media exposure according to their needs. Our work supports the call for new approaches to understanding media effects, specifically those that acknowledge media use as a dynamic, human adaptation to the environment [14, 26] where both genes and environments play an integral roll.

A limitation of this study, besides the general limitations of the twin method [12], is that media use was assessed with reference to home computers only. In 2012 the first 4G network became available in the UK (http://www.ons.gov.uk/ons/rel/rdit2/internet-access---households-and-individuals/2013/stb-ia-2013.html#tab-Mobile-Internet) leading to an exponential increase in use of mobile phones and other portable devices. However, substantial genetic influence has been demonstrated for mobile phone use, including time spent talking on the phone (34–60%) and frequency of sending text messages (50–53%;[11]), in line with our current results.

### Conclusion

Online media use provides an excellent opportunity to investigate genetic contributions to experience, which result from individuals' active selection of environmental engagements influenced in part by the individuals' genetic propensities. As access to and engagement with online media continues to grow at an unprecedented speed (http://www.ons.gov.uk/ons/dcp171778_404497.pdf), it plays an increasingly important role for the development and experience of people across all age groups and thus, becomes an even more valuable subject of study. Our current study, which is one of the first investigations in this area, suggests that roughly one third of the variance in online media use can be attributed to genetic influences. We predict that future studies will find even greater heritability estimates, as online media continue to permeate our environment and as media use is tailored even more to our personal needs and interests. As environmental differences in access and availability diminish, our data suggests that differences in online media use would increasingly reflect differences in genetic propensities as individuals choose to use online media in line with their genetic propensities.

## Supporting Information

S1 Fig Portion of questionnaire assessing online media use. (DOCX)

S2 Fig Portion of questionnaire assessing Facebook use. (DOCX)

S1 Table Proportion of time spent on different forms of media for total sample and across gender and zygosity. (DOCX)

S2 Table Factor analyses on media use variables. (DOCX)

S3 Table Total variance explained in media use factor analysis. (DOCX)

S4 Table Factor analyses on Facebook use variables. (DOCX)

S5 Table Total variance explained in Facebook factor analysis. (DOCX)

S6 Table Sex limitation model fitting results with 95% confidence intervals, depicting A, C and E estimates separately for males and females. (DOCX)

S7 Table Sex limitation sub-model comparisons: Factorized entertainment screen time. (DOCX)

S8 Table Sex limitation sub-model comparisons: Factorized educational screen time. (DOCX)

S9 Table Sex limitation sub-model comparisons: Factorized gaming. (DOCX)

S10 Table Sex limitation sub-model comparisons: Facebook Factor. (DOCX)

S11 Table Univariate genetic results. (DOCX)
