# Supplementary figures and images for: Personalized Media: A Genetically Informative Investigation of Individual Differences in Online Media Use
Source: PLoS One. 2017 Jan 23;12(1):e0168895. doi: 10.1371/journal.pone.0168895 (PMC5256859; doi:10.1371/journal.pone.0168895)

**Figure S1.** Portion of questionnaire assessing online media use


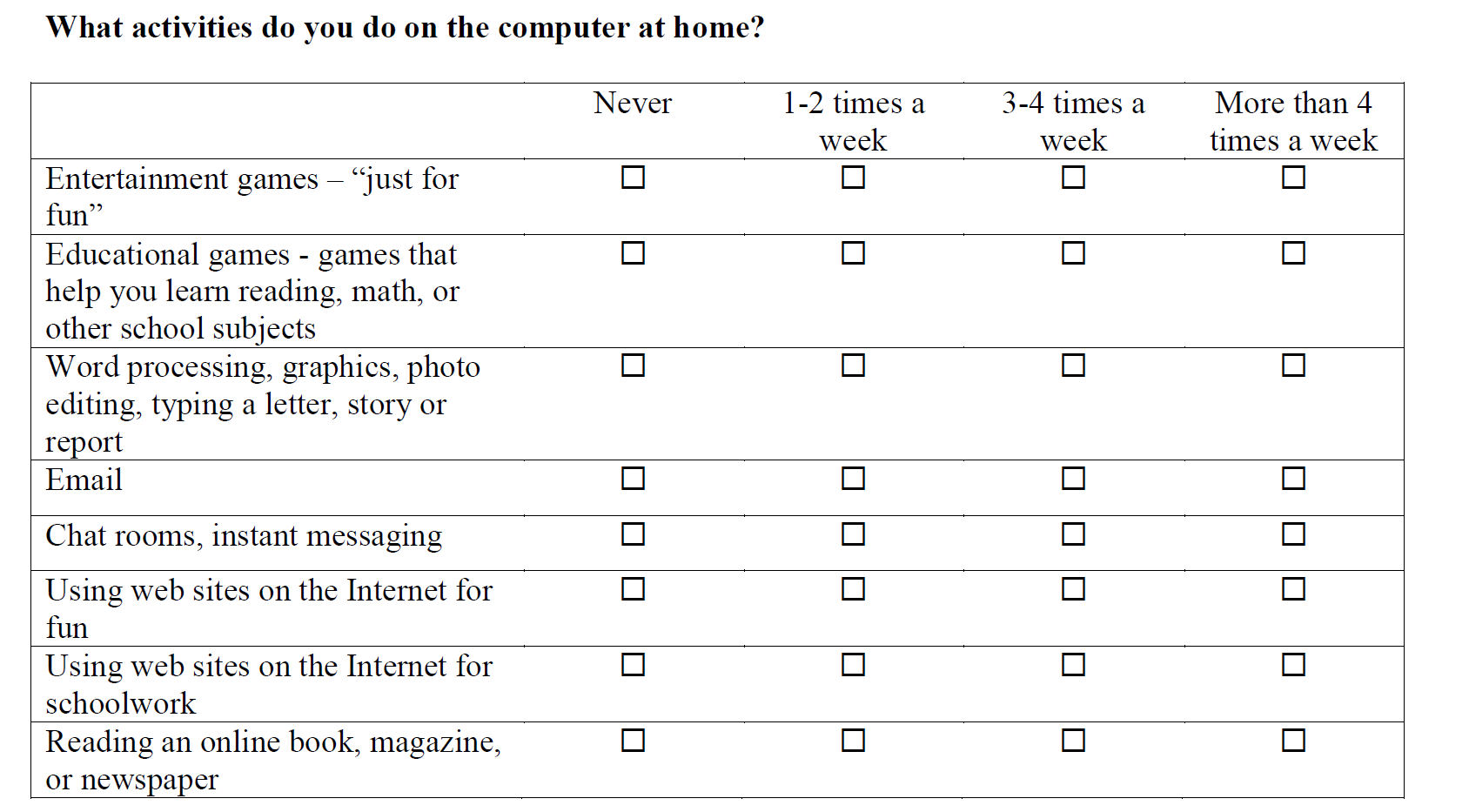

Supplement: S1 Fig — (DOCX) [file pone.0168895.s001.docx]

**Figure S2**. Portion of questionnaire assessing Facebook use


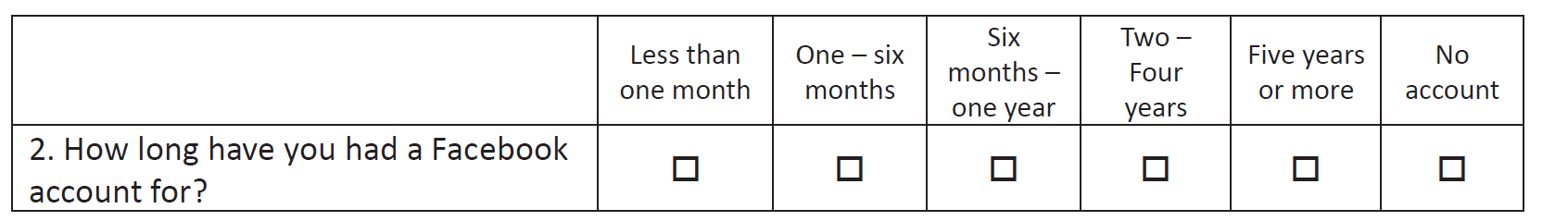


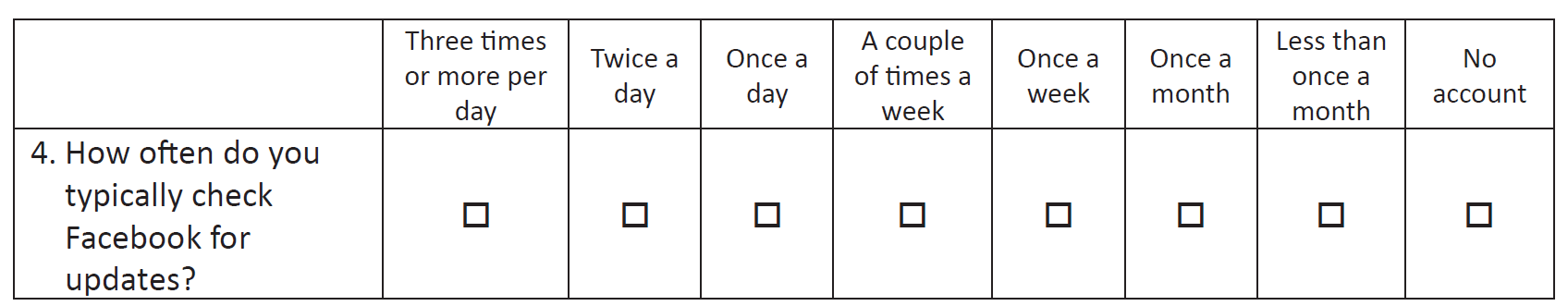


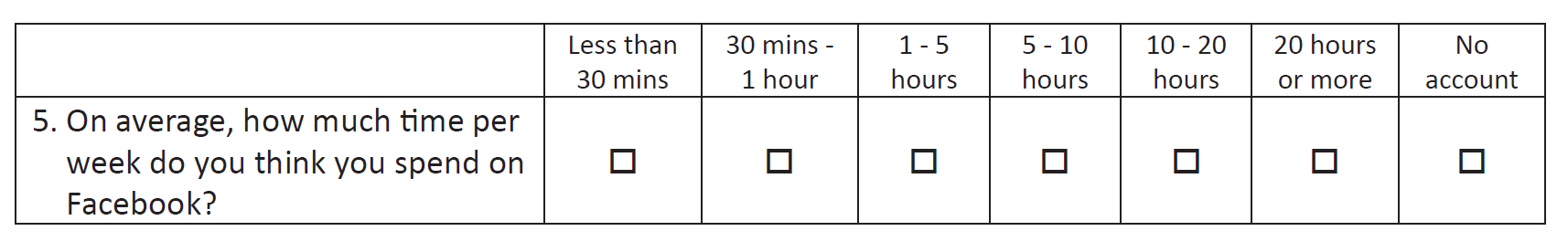


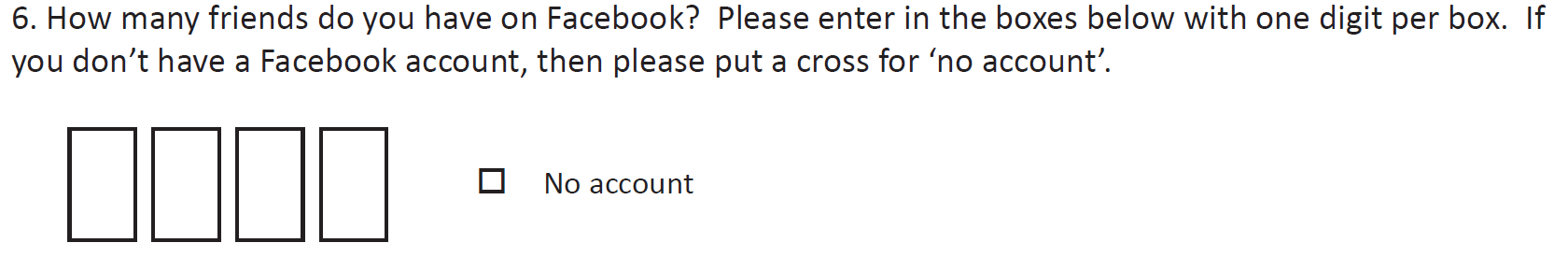

Supplement: S2 Fig — (DOCX) [file pone.0168895.s002.docx]
