# Supplementary material for: Personalized Media: A Genetically Informative Investigation of Individual Differences in Online Media Use
Source: PLoS One. 2017 Jan 23;12(1):e0168895. doi: 10.1371/journal.pone.0168895 (PMC5256859; doi:10.1371/journal.pone.0168895)
Supplement: S1 Table — (DOCX) [file pone.0168895.s003.docx]

**Table S1.** Mean (standard deviation**)** time spent on different forms of media for the total sample and across gender and zygosity. N= sample size after exclusions (individuals); MZ= monozygotic; DZ= dizygotic; m= male; f= female; os= opposite sex, Entertain= time spent on entertainment media, Educate= time spent on educational media, Gaming= time spent on online games for fun or for educational purposes, Facebook= level of engagement with the social network Facebook. ANOVA performed on raw data from one randomly selected twin per pair to test the effect of sex and zygosity. Results = F statistic; * = p<.05; ** = p<.01; R^2^= proportion of variance explained by sex, zygosity and their interaction.

|  |  | **Whole** |  |  |  |  |  |  |  |  |  |  |  |  |
| --- | --- | --- | --- | --- | --- | --- | --- | --- | --- | --- | --- | --- | --- | --- |
| **Media Use** | **N** | **Sample** | **Male** | **Female** | **MZm** | **DZm** | **MZf** | **DZf** | **Dzos** | **DZss** | **Sex** | **Zyg** | **Sex x Zyg** | **R2** |
| Entertain | 5221 | 5.84 | 5.87 | 5.83 | 5.85 | 5.86 | 5.71 | 5.84 | 5.94 | 5.85 | 0.73 | 6.78** | 3.07* | <.01 |
|  |  | (1.69) | (1.67) | (1.72) | (1.72) | (1.67) | (1.75) | (1.69) | (1.66) | (1.68) |  |  |  |  |
| Educate | 5181 | 9.15 | 8.96 | 9.28 | 8.97 | 8.88 | 9.24 | 9.20 | 9.25 | 9.07 | 18.77** | 0.06 | 3.70** | <.01 |
|  |  | (2.62) | (2.70) | (2.56) | (2.74) | (2.73) | (2.50) | (2.56) | (2.64) | (2.64) |  |  |  |  |
| Gaming | 4273 | 3.44 | 3.90 | 3.11 | 4.01 | 3.82 | 3.08 | 3.14 | 3.46 | 3.42 | 521.49** | 0.00 | 93.56** | 0.07 |
|  |  | (1.30) | (1.32) | (1.19) | (1.37) | (1.29) | (1.18) | (1.18) | (1.30) | (1.27) |  |  |  |  |
| Facebook | 8648 | 0.00 | -0.06 | 0.06 | -0.11 | -0.04 | 0.03 | 0.05 | 0.02 | 0.01 | 33.30** | 3.27 | 6.63** | <.01 |
|  |  | (0.96) | (1.04) | (0.88) | (0.99) | (1.10) | (0.88) | (0.95) | (0.93) | (1.01) |  |  |  |  |

*Note.* All means and standard deviations are reported after factor analyses, for Facebook use the measures were also standardized
