# Supplementary material for: Personalized Media: A Genetically Informative Investigation of Individual Differences in Online Media Use
Source: PLoS One. 2017 Jan 23;12(1):e0168895. doi: 10.1371/journal.pone.0168895 (PMC5256859; doi:10.1371/journal.pone.0168895)
Supplement: S2 Table — (DOCX) [file pone.0168895.s004.docx]

**Table S2.** Factor analyses on media use variables

|  | Factors | | |
| --- | --- | --- | --- |
|  | 1 | 2 | 3 |
| Websites for School | .766 |  |  |
| Word Processing | .757 | .113 |  |
| Reading Online | .500 |  | .257 |
| Chatrooms |  | .762 |  |
| Websites for Fun |  | .602 | .416 |
| Email | .485 | .515 |  |
| Entertainment Games |  | .210 | .820 |
| Educational Games | .411 | -.329 | .551 |
