# Supplementary material for: Personalized Media: A Genetically Informative Investigation of Individual Differences in Online Media Use
Source: PLoS One. 2017 Jan 23;12(1):e0168895. doi: 10.1371/journal.pone.0168895 (PMC5256859; doi:10.1371/journal.pone.0168895)
Supplement: S3 Table — (DOCX) [file pone.0168895.s005.docx]

**Table S3.** Total variance explained in media use factor analysis

|  | Initial Eigenvalues | | | Extraction Sums of Squared Loadings | | | Rotation Sums of Squared Loadings | | |
| --- | --- | --- | --- | --- | --- | --- | --- | --- | --- |
| Component | Total | % of Variance | Cumulative % | Total | % of Variance | Cumulative % | Total | % of Variance | Cumulative % |
| 1 | 2.089 | 26.109 | 26.109 | 2.089 | 26.109 | 26.109 | 1.871 | 23.384 | 23.384 |
| 2 | 1.254 | 15.669 | 41.778 | 1.254 | 15.669 | 41.778 | 1.344 | 16.797 | 40.181 |
| 3 | 1.115 | 13.938 | 55.716 | 1.115 | 13.938 | 55.716 | 1.243 | 15.534 | 55.716 |
| 4 | .883 | 11.042 | 66.758 |  |  |  |  |  |  |
| 5 | .763 | 9.535 | 76.293 |  |  |  |  |  |  |
| 6 | .739 | 9.243 | 85.536 |  |  |  |  |  |  |
| 7 | .628 | 7.847 | 93.383 |  |  |  |  |  |  |
| 8 | .529 | 6.617 | 100.000 |  |  |  |  |  |  |
