# Supplementary material for: Personalized Media: A Genetically Informative Investigation of Individual Differences in Online Media Use
Source: PLoS One. 2017 Jan 23;12(1):e0168895. doi: 10.1371/journal.pone.0168895 (PMC5256859; doi:10.1371/journal.pone.0168895)
Supplement: S4 Table — (DOCX) [file pone.0168895.s006.docx]

**Table S4.** Factor analyses on Facebook use variables.

|  | Factors |
| --- | --- |
|  | 1 |
| How often check updates | .813 |
| How much time spent per week | .811 |
| How long had account | .492 |
| How many Facebook friends | .486 |
