# Supplementary material for: Personalized Media: A Genetically Informative Investigation of Individual Differences in Online Media Use
Source: PLoS One. 2017 Jan 23;12(1):e0168895. doi: 10.1371/journal.pone.0168895 (PMC5256859; doi:10.1371/journal.pone.0168895)
Supplement: S5 Table — (DOCX) [file pone.0168895.s007.docx]

**Table S5.** Total variance explained in Facebook factor analysis

|  | Initial Eigenvalues | | | Extraction Sums of Squared Loadings | | |
| --- | --- | --- | --- | --- | --- | --- |
| Component | Total | % of Variance | Cumulative % | Total | % of Variance | Cumulative % |
| 1 | 1.796 | 44.890 | 44.890 | 1.796 | 44.890 | 44.890 |
| 2 | .960 | 24.006 | 68.896 |  |  |  |
| 3 | .818 | 20.462 | 89.358 |  |  |  |
| 4 | .426 | 10.642 | 100.000 |  |  |  |
