# Supplementary material for: Personalized Media: A Genetically Informative Investigation of Individual Differences in Online Media Use
Source: PLoS One. 2017 Jan 23;12(1):e0168895. doi: 10.1371/journal.pone.0168895 (PMC5256859; doi:10.1371/journal.pone.0168895)
Supplement: S6 Table — (DOCX) [file pone.0168895.s008.docx]

**Table S6.** Sex limitation results for additive genetic (A), shared environmental (C) and non-shared environmental

(C) components of variance (including error), with 95% confidence intervals A, C and E estimates (95% confidence intervals)

|  | Male | | | Female | | |
| --- | --- | --- | --- | --- | --- | --- |
|  | **A** | **C** | **E** | **A** | **C** | **E** |
| Entertain | 0.35 | 0.15 | 0.51 | 0.36 | 0.09 | 0.55 |
|  | (0.18-0.50) | (0.02-0.29) | (0.46-0.56) | (0.21-0.47) | (0.01-0.22) | (0.50-0.59) |
|  |  |  |  |  |  |  |
| Educate | 0.35 | 0.06 | 0.59 | 0.34 | 0.09 | 0.57 |
|  | (0.20-0.45) | (0.00-0.18) | (0.53-0.65) | (0.21-0.45) | (0.00-0.20) | (0.53-0.61) |
|  |  |  |  |  |  |  |
| Gaming | 0.39 | 0.00 | 0.61 | 0.23 | 0.15 | 0.61 |
|  | (0.22-0.44) | (0.00-0.14) | (0.56-0.66) | (0.08-0.39) | (0.02-0.28) | (0.57-0.66) |
|  |  |  |  |  |  |  |
| Facebook | 0.43 | 0.36 | 0.21 | 0.48 | 0.32 | 0.20 |
|  | (0.32-0.54) | (0.26-0.47) | (0.18-0.24) | (0.38-0.57) | (0.23-0.41) | (0.18-0.22) |
