# Supplementary material for: Personalized Media: A Genetically Informative Investigation of Individual Differences in Online Media Use
Source: PLoS One. 2017 Jan 23;12(1):e0168895. doi: 10.1371/journal.pone.0168895 (PMC5256859; doi:10.1371/journal.pone.0168895)
Supplement: S7 Table — (DOCX) [file pone.0168895.s009.docx]

**Table S7** Sex limitation sub-model comparisons: Factorized entertainment screen time

| **Model** | **ep** | **X^2^** | **df** | **AIC** | **∆ X^2^** | **∆ df** | ***p*** |
| --- | --- | --- | --- | --- | --- | --- | --- |
| Full sex-limited | 9 | 29906.13 | 10789 | 8328.13 | - | - | - |
| Qualitative (fixed rG) | 8 | 29906.29 | 10790 | 8326.29 | 0.16 | 1 | 0.69 |
| Qualitative (fixed rC) | 8 | 29906.29 | 10790 | 8326.29 | 0.16 | 1 | 0.69 |
| Quantitative genetic | 5 | 29918.31 | 10793 | 8332.31 | 12.02 | 3 | <.01 |
