# Supplementary material for: Personalized Media: A Genetically Informative Investigation of Individual Differences in Online Media Use
Source: PLoS One. 2017 Jan 23;12(1):e0168895. doi: 10.1371/journal.pone.0168895 (PMC5256859; doi:10.1371/journal.pone.0168895)
Supplement: S8 Table — (DOCX) [file pone.0168895.s010.docx]

**Table S8.** Sex limitation sub-model comparisons: Factorized educational screen time

| **Model** | **ep** | **X^2^** | **df** | **AIC** | **∆ X^2^** | **∆ df** | ***p*** |
| --- | --- | --- | --- | --- | --- | --- | --- |
| Full sex-limited | 9 | 30250.86 | 10871 | 8508.86 | - | - | - |
| Qualitative (fixed rG) | 8 | 30250.86 | 10872 | 8506.86 | 0 | 1 | 1.00 |
| Qualitative (fixed rC) | 8 | 30250.86 | 10872 | 8506.86 | 0 | 1 | 1.00 |
| Quantitative genetic | 5 | 30260.14 | 10875 | 8510.14 | 9.28 | 3 | 0.03 |
