# Supplementary material for: Personalized Media: A Genetically Informative Investigation of Individual Differences in Online Media Use
Source: PLoS One. 2017 Jan 23;12(1):e0168895. doi: 10.1371/journal.pone.0168895 (PMC5256859; doi:10.1371/journal.pone.0168895)
Supplement: S9 Table — (DOCX) [file pone.0168895.s011.docx]

**Table S9**. Sex limitation sub-model comparisons: Factorized gaming

| **Model** | **ep** | **X^2^** | **df** | **AIC** | **∆ X^2^** | **∆ df** | ***p*** |
| --- | --- | --- | --- | --- | --- | --- | --- |
| Full sex-limited | 9 | 30520.10 | 10983 | 8554.10 | - | - | - |
| Qualitative (fixed rG) | 8 | 30520.10 | 10984 | 8552.10 | 0 | 1 | 1.00 |
| Qualitative (fixed rC) | 8 | 30520.10 | 10984 | 8552.10 | 0 | 1 | 1.00 |
| Quantitative genetic | 5 | 30682.32 | 10987 | 8708.32 | 162.21 | 3 | <.01 |
