# Supplementary material for: Personalized Media: A Genetically Informative Investigation of Individual Differences in Online Media Use
Source: PLoS One. 2017 Jan 23;12(1):e0168895. doi: 10.1371/journal.pone.0168895 (PMC5256859; doi:10.1371/journal.pone.0168895)
Supplement: S10 Table — (DOCX) [file pone.0168895.s012.docx]

**Table S10**. Sex limitation sub-model comparisons: Facebook Factor

| **Model** | **ep** | **X^2^** | **df** | **AIC** | **∆ X^2^** | **∆ df** | ***p*** |
| --- | --- | --- | --- | --- | --- | --- | --- |
| Full sex-limited | 9 | 50438.69 | 18121 | 14196.69 | - | - | - |
| Qualitative (fixed rG) | 8 | 50438.69 | 18122 | 14194.69 | -2.619345^e-10^ | 1 | 1.00 |
| Qualitative (fixed rC) | 8 | 50438.69 | 18122 | 14194.69 | -2.692104^e-10^ | 1 | 1.00 |
| Quantitative genetic | 5 | 50438.69 | 18125 | 14188.69 | -2.182787^e-11^ | 3 | 1.00 |
