# Supplementary material for: Personalized Media: A Genetically Informative Investigation of Individual Differences in Online Media Use
Source: PLoS One. 2017 Jan 23;12(1):e0168895. doi: 10.1371/journal.pone.0168895 (PMC5256859; doi:10.1371/journal.pone.0168895)
Supplement: S11 Table — (DOCX) [file pone.0168895.s013.docx]

**Table S11**. Univariate genetic results for additive genetic (A), shared environmental (C) and

non-shared environmental (E) components of variance (including error), with 95% confidence intervals.

|  |  |  |  | Intraclass correlations | |
| --- | --- | --- | --- | --- | --- |
|  | A | C | E | MZ | DZ |
| Entertainment sites | 0.37 | 0.10 | 0.53 | 0.45 | 0.30 |
|  | (0.27-0.46) | (0.05-0.18) | (0.50-0.56) |  |  |
| Educational sites | 0.34 | 0.08 | 0.58 | 0.44 | 0.24 |
|  | (0.25-0.44) | (0.01-0.15) | (0.54-0.61) |  |  |
| Gaming | 0.39 | 0.00 | 0.60 | 0.40 | 0.20 |
|  | (0.29-0.43) | (0-0.08) | (0.57-0.64) |  |  |
| Facebook | 0.24 | 0.20 | 0.56 | 0.41 | 0.33 |
|  | (0.17-0.32) | (0.14-0.25) | (0.53-0.59) |  |  |
